# Strategies for prolonging ventricular action potential duration without increasing transmural dispersion of repolarization

**DOI:** 10.14814/phy2.70693

**Published:** 2025-12-05

**Authors:** Candido Cabo

**Affiliations:** ^1^ Department of Computer Systems New York City College of Technology New York New York USA; ^2^ Graduate Center City University of New York New York New York USA

**Keywords:** computer models, dispersion of repolarization, ion channels, multichannel pharmacology

## Abstract

Dispersion of repolarization results from a non‐homogeneous recovery of excitability in cardiac tissue, and it is an important factor in arrhythmogenesis because it could lead to the initiation and maintenance of a variety of arrhythmias. Antiarrhythmic agents that prolong action potential duration (APD) by selectively blocking specific ion channels (like I_Kr_) often increase dispersion of repolarization, which could result in a pro‐arrhythmic risk. In this report, using computer models of the action potential of human epicardial, mid‐myocardial, and endocardial myocytes, we have identified strategies to prolong APD without increasing transmural dispersion of repolarization. The first strategy, which involves blocking several depolarizing and repolarizing ion channels (I_NaL_, I_CaL_, I_Kr_, and I_NaCa_), can prolong APD while decreasing transmural APD dispersion by about 20%–60%, depending on the model. The second strategy, which involves the use of a combination of ion channel blockers and activators, can prolong APD while decreasing transmural APD dispersion by about 70%, a stronger reduction in transmural dispersion of repolarization than using only ion channel blockers. Our results suggest that a multichannel pharmacology strategy (as opposed to a single channel strategy), possibly using ion channel blockers and activators, can be effective at increasing APD while minimizing dispersion of repolarization.

## 1 INTRODUCTION

Dispersion of repolarization results from a non‐homogeneous recovery of excitability in cardiac tissue and can be influenced by various natural and pathological conditions (Antzelevitch, 2007, 2008; Lukas, 1997; Surawicz, 1989). Cells in the epicardial, mid‐myocardial, and endocardial layers of the ventricles differ in their electrophysiological characteristics and their response to pharmacological agents (Antzelevitch, 2008; Lukas, 1997). In normal human hearts, dispersion of repolarization is generally small and considered benign (Kang et al., 2017). However, there are genetic factors, acquired pathological conditions as well as unintended effects from antiarrhythmic drugs that may exacerbate the naturally occurring dispersion of repolarization (Antzelevitch, 2007; Antzelevitch, 2008; Shimizu & Antzelevitch, 1998).

Dispersion of repolarization is an important factor in arrhythmogenesis, and it could lead to the initiation and maintenance of a variety of arrhythmias including Torsade de Pointes (TdP) and atrial fibrillation (AF) (Antzelevitch, 2005, 2008; Avula et al., 2019; Kuo et al., 1985; Surawicz, 1989). Increased transmural dispersion of repolarization, frequently due to preferential prolongation of the APD of mid‐myocardial cells, provides the arrhythmogenic substrate for TdP in patients with acquired or congenital LQT syndrome (Antzelevitch, 2005, 2008; Surawicz, 1989). In that substrate, a premature ventricular contraction could lead to unidirectional block and initiation of reentrant waves (Antzelevitch, 2005, 2007; Belardinelli et al., 2003). Paroxysmal or persistent AF results in spatial heterogeneities in APD in human patients (Avula et al., 2019; Diker et al., 1998; Li et al., 2001), suggesting that dispersion of repolarization may provide a substrate for the initiation and maintenance of AF (Avula et al., 2019).

Antiarrhythmic agents can have a proarrhythmic risk if they increase dispersion of repolarization. Class I antiarrhythmic drugs block the sodium channel but can also affect other ion channels, and they may increase or decrease dispersion of repolarization depending on their specific mechanism of action (Belardinelli et al., 2003; Surawicz, 1989). Class IA drugs, like quinidine and procainamide, block both sodium and potassium channels prolonging APD and are generally associated with an increased dispersion of repolarization and a higher risk of TdP (Antzelevitch, 2008; Surawicz, 1989), but not always (Milberg et al., 2007). Class IB agent mexiletine blocks inactivated sodium channels and reduces both APD and dispersion of repolarization (Shimizu & Antzelevitch, 1997). Class IC agent flecainide, similarly to some Class IA agents, tends to increase dispersion and contribute to arrhythmogenesis (Antzelevitch, 2008). The impact of these drugs on dispersion of repolarization is a critical factor in their proarrhythmic risk (Antzelevitch, 2005).

Class III antiarrhythmic drugs primarily act by blocking potassium channels, leading to APD prolongation to prevent reentrant arrhythmias (Peters et al., 2000). Many Class III antiarrhythmic drugs, like sotalol and dofetilide, which act by blocking the rapid delayed rectifier ion channel (I_Kr_), tend to increase dispersion of repolarization by preferentially prolonging the APD of mid‐myocardial cells (Antzelevitch, 2008; Lukas, 1997) and creating a substrate for reentrant arrhythmias like TdP (Antzelevitch, 2005). However, there are class III agents that prolong APD homogeneously without an increase in dispersion of repolarization. For example, chromanol 293B, which blocks the slow delayed rectifier current (I_Ks_), prolongs APD homogenously without an increase in dispersion of repolarization (Antzelevitch, 2005, 2007). Amiodarone, a multi‐channel blocker often classified as a class III antiarrhythmic agent, has been shown to increase APD while reducing dispersion of repolarization by prolonging APD in endocardial and epicardial cells but not in mid‐myocardial cells (Árpádffy‐Lovas et al., 2021; Drouin et al., 1998; Gelman et al., 2024; Sicouri et al., 1997; Vassallo & Trohman, 2007).

Other heart‐acting drugs like anti‐anginal and adrenergic agents may also cause a decrease in the dispersion of repolarization. The anti‐anginal agent ranolazine, which acts on sodium, calcium and potassium channels, has a similar electrophysiological effect to amiodarone by prolonging APD in epicardial cells but not in mid‐myocardial cells, and therefore reducing the dispersion of repolarization (Antzelevitch et al., 2004; Hasenfuss & Maier, 2008). Carvedilol is an alpha‐ and beta‐adrenergic antagonist that also modulates potassium, sodium, and calcium channels (Karle et al., 2001). In a rabbit model of congestive heart failure, carvedilol causes a reduction in the dispersion of repolarization by prolonging APD in epicardial and endocardial cells to a larger extent than in mid‐myocardial cells (Zhong et al., 2007). Anesthetics like sodium pentobarbital and propofol have also been shown to reduce the dispersion of repolarization by prolonging APD in epicardial and endocardial cells to a larger extent than in mid‐myocardial cells (Ellermann et al., 2020; Shimizu et al., 1999).

In summary, while APD prolongation often results in an increased dispersion of repolarization, it is the increase in dispersion itself that is considered the primary arrhythmogenic substrate for TdP (Antzelevitch, 2005, 2008; Árpádffy‐Lovas et al., 2021; Belardinelli et al., 2003). Pharmacological agents that decrease dispersion of repolarization (like amiodarone, ranolazine, and pentobarbital) often achieve this through the modulation of multiple ion channels, by having a differential effect in different myocardial cell types resulting in a more homogeneous recovery of excitability. This reduction in dispersion is considered crucial for mitigating the risk of serious arrhythmias (Antzelevitch et al., 2004; Antzelevitch, 2005, 2007, 2008; Trenor et al., 2013; Árpádffy‐Lovas et al., 2021; Ellermann et al., 2020). In this report, using computer models of the human ventricle, we investigate: (1) mechanisms by which multi‐channel pharmacology can reduce transmural dispersion of repolarization; (2) strategies that could prolong APD without an increase in transmural dispersion of repolarization.

## 2 METHODS

### 2.1 Computer models of the action potential

We simulated the cardiac action potential using the ORd (O'Hara et al., 2011) models of human ventricular epicardial, mid‐myocardial and endocardial cells. The models are publicly available from the Rudy Lab web site (https://rudylab.wustl.edu/code‐downloads/). We also used the ToR‐ORd model (Tomek et al., 2019), which was downloaded from the CellML repository (www.cellml.org). The ToR‐ORd model builds on the structure of the ORd model, but the formulation of several depolarizing and repolarizing currents, like I_CaL_, I_Kr_, and I_K1_, is different (Tomek et al., 2019). We estimated transmural dispersion of repolarization as the maximum difference between APDs between epicardial, mid‐myocardial and endocardial cells, while recognizing that in myocardial tissue transmural dispersion of repolarization is also affected by cell‐to‐cell coupling, cardiac conduction and APD gradients (Glukhov et al., 2010).

We investigated changes in transmural dispersion of repolarization between epicardial, mid‐myocardial and endocardial action potential models using ion channel blockers and enhancers (activators) by modulating the maximum conductance of: late sodium current (I_NaL_; range: 0–2× control), L‐type calcium current (I_CaL_; range: 0.5–1.5× control), slow delayed rectifier potassium current (I_Ks_; range: 0–50× control), rapid delayed rectifier potassium current (I_Kr_; range: 0–2× control), inward rectifier potassium current (I_K1_; range: 0.2–2× control), sodium/potassium pump (I_NaK_; range: 0.5–1.5× control) and sodium/calcium exchanger (I_NaCa_; range: 0.5–1.5× control). For the simulations using only ion channel blockers we limited the maximum conductance of the ion channel to that of control. The ranges of variations of maximum conductance for the different ion channels were selected to avoid the occurrence of early afterdepolarizations and other repolarization abnormalities in the action potentials generated by the models. Action potentials were initiated with a depolarizing current with a strength 1.5× the stimulation threshold. We report measurements on action potentials that were calculated after 30 min of stimulation to achieve steady state (Cabo, 2022).

### 2.2 Action potential features

The phases of the action potential were quantified as described in an earlier report (Cabo, 2022). In short, phase 1 begins at the end of action potential depolarization and it ends at the time repolarization starts, which is when the total ion current becomes positive (Figure 1 in Cabo, 2022). Phase 2 starts when phase 1 ends, and it ends when I_K1_ rises to 10% of its peak (Cabo, 2022). In the ORd model the end of phase 2 occurs when the membrane repolarizes to −39 mV. In the ToR‐ORd model the end of phase 2 occurs when the membrane repolarizes to −34 mV. Phase 3 starts at the end of phase 2, and it ends when the action potential repolarizes by 90% of the action potential amplitude from its maximum depolarization potential. The action potential amplitude (APA) is the difference between the maximum depolarization potential (V_m,peak_) and the resting membrane potential (V_m,rest_). Action potential duration (APD) is defined as the interval between the time of depolarization and the time at which the action potential repolarizes by 90% of the APA from V_m,peak_ (i.e., APD = phase 1 + phase 2 + phase 3) (Figure 1 in Cabo, 2022).

### 2.3 Estimation of the repolarization reserve

As before (Cabo, 2022), we estimated the repolarization reserve of a baseline action potential by quantifying the prolongation of the APD upon application of a constant depolarizing current of −0.1pA/pF during the action potential (Varro & Baczko, 2011). This can be done experimentally for example by increasing the late sodium current (I_NaL_) with veratrine and anemonia sulcata toxin (ATX II; Varro & Baczko, 2011). With that protocol, a larger prolongation of APD with respect to the baseline APD implies a smaller repolarization reserve and a higher risk of triggered arrhythmias.

### 2.4 Particle swarm optimization algorithm

As before (Cabo, 2022), we used the particle swarm optimization (PSO) algorithm (Kennedy & Eberhart, 1995) to find the optimal combination of maximum conductance of I_NaL_, I_CaL_, I_Ks_, I_Kr_, I_K1_ I_NaK_, and I_NaCa_ to minimize the difference between APD in epicardial and mid‐myocardial cells. The PSO algorithm works by having a population (swarm) of particles (candidate solutions) search a parameter space. The algorithm starts with a randomly generated position (a possible solution) and velocity for each particle. In each iteration, each particle evaluates its solution based on a function goal, updates its particle's best solution (pbest), and if pbest is better than the global best solution (gbest), then gbest is updated. Each particle then updates its velocity based on pbest and gbest balanced by an inertia weight strategy. The velocity is used to calculate a new position of each particle (solution). To avoid getting trapped in a local minimum, particles exchange information only with a subset of particles (neighborhood size) in the swarm. The PSO algorithm is a heuristic algorithm that does not guarantee that the optimal global solution is found. We used the following PSO algorithm settings: number of particles in the swarm (20); neighborhood topology (ring); neighborhood size (10); linearly decreasing inertia weight strategy. In our simulations, after 50 iterations, the swarm converged to a solution that minimized the goal. We used an implementation of the PSO algorithm publicly available in the GitHub repository (https://github.com/kkentzo/pso).

The goal of the PSO optimization algorithm was to minimize the APD differences between epicardial and mid‐myocardial cells, and differences between the APD of either epicardial or mid‐myocardial cells and a given APD target set by the user, depending on the optimization simulation. The ion currents and the range of variation (minimum and maximum values) of ion channel maximum conductance allowed for the optimization process are specified above in section *Computer Models of the Action Potential*. Combinations of ion channel maximum conductances that resulted in early afterdepolarizations or other repolarization abnormalities were discarded.

### 2.5 Backward feature elimination

We used a backward feature elimination procedure to investigate the relative contribution of each ion current to the transmural heterogeneities (dispersion) in the action potential (Cabo, 2023). After applying the PSO optimization to find the combination of maximum conductance of I_NaL_, I_CaL_, I_Ks_, I_Kr_, I_K1_, I_NaK_, and I_NaCa_ that minimize APD dispersion, optimization was applied to the seven possible subsets of six currents (i.e., [I_CaL_, I_Ks_, I_Kr_, I_K1_, I_NaK_, and I_NaCa_], [I_NaL_, I_Ks_, I_Kr_, I_K1_, I_NaK_, and I_NaCa_], [I_NaL_, I_Ca_, I_Kr_, I_K1_, I_NaK_, and I_NaCa_], [I_NaL_, I_CaL_, I_Ks_, I_K1_, I_NaK_, and I_NaCa_], [I_NaL_, I_CaL_, I_Ks_, I_Kr_, I_NaK_, and I_NaCa_], [I_NaL_, I_CaL_, I_Ks_, I_Kr_, I_K1_, and I_NaCa_], [I_NaL_, I_CaL_, I_Ks_, I_Kr_, I_K1_, and I_NaK_]). The current not present in each subset was kept at the control value. The subset resulting in the larger reduction of dispersion after PSO optimization was selected for the next step in the elimination procedure. The ion current not present in the selected subset was the current that contributed less to a reduction in APD dispersion, and it was consequently eliminated (i.e., its maximum conductance was set to the control value). This process of elimination was repeated until only two ion currents were left.

## 3 RESULTS

### 3.1 Transmural action potential heterogeneity

**FIGURE 1:**
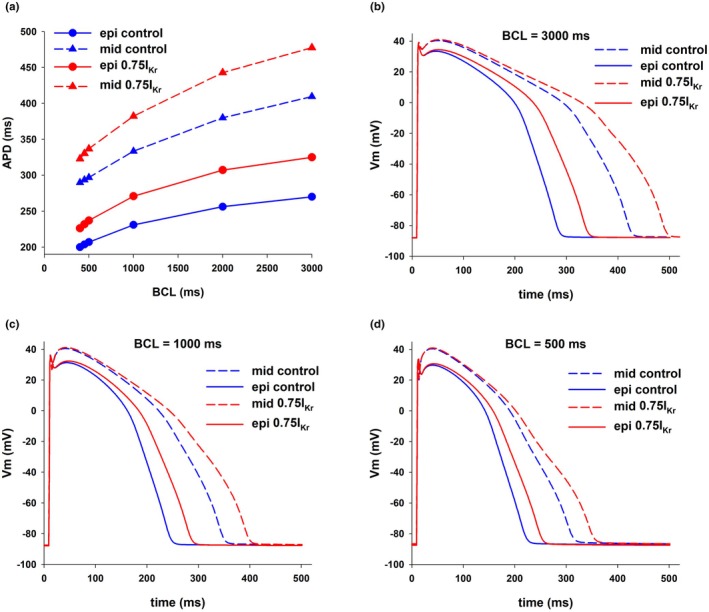
Dispersion of repolarization between epicardial and mid‐myocardial cells. (a) Action potential duration (APD) during control (blue) and 25% block of I_Kr_ (red), for epicardial (circles, solid lines) and mid‐myocardial cells (triangles, dashed lines), for different BCLs. (b) Action potentials for BCL = 3000 ms. (c) Action potentials for BCL = 1000 ms. (d) Action potentials for BCL = 500 ms. See text for detailed description.

Figure 1a shows the differences in APD between epicardial (solid blue circles; solid blue line) and mid‐myocardial (solid blue triangles; dashed blue line) cells, for BCLs between 400 and 3000 ms under physiological conditions (control) in the ORd model. For each BCL, APD of mid‐myocardial cells is larger than APD in epicardial cells. Differences in APD (transmural APD dispersion) range from 139 ms at BCL = 3000–90 ms at BCL = 400 ms (Figure 1a). Figure 1a also shows that selective block of I_Kr_ increases APD dispersion by increasing APD in mid‐myocardial cells to a larger extent than APD in epicardial cells (epicardial cells: red circles, solid red line; mid‐myocardial cells: red triangles, dashed red line). With 25% I_Kr_ block, APD dispersion increased from 139 to 152 ms at BCL = 3000 ms (~9% increase), and from 90 to 97 ms at BCL = 400 ms (~8% increase). Action potentials for epicardial and mid‐myocardial cells during control and 25% I_Kr_ block for three BCLs are shown in Figure 1b–d.

**FIGURE 2:**
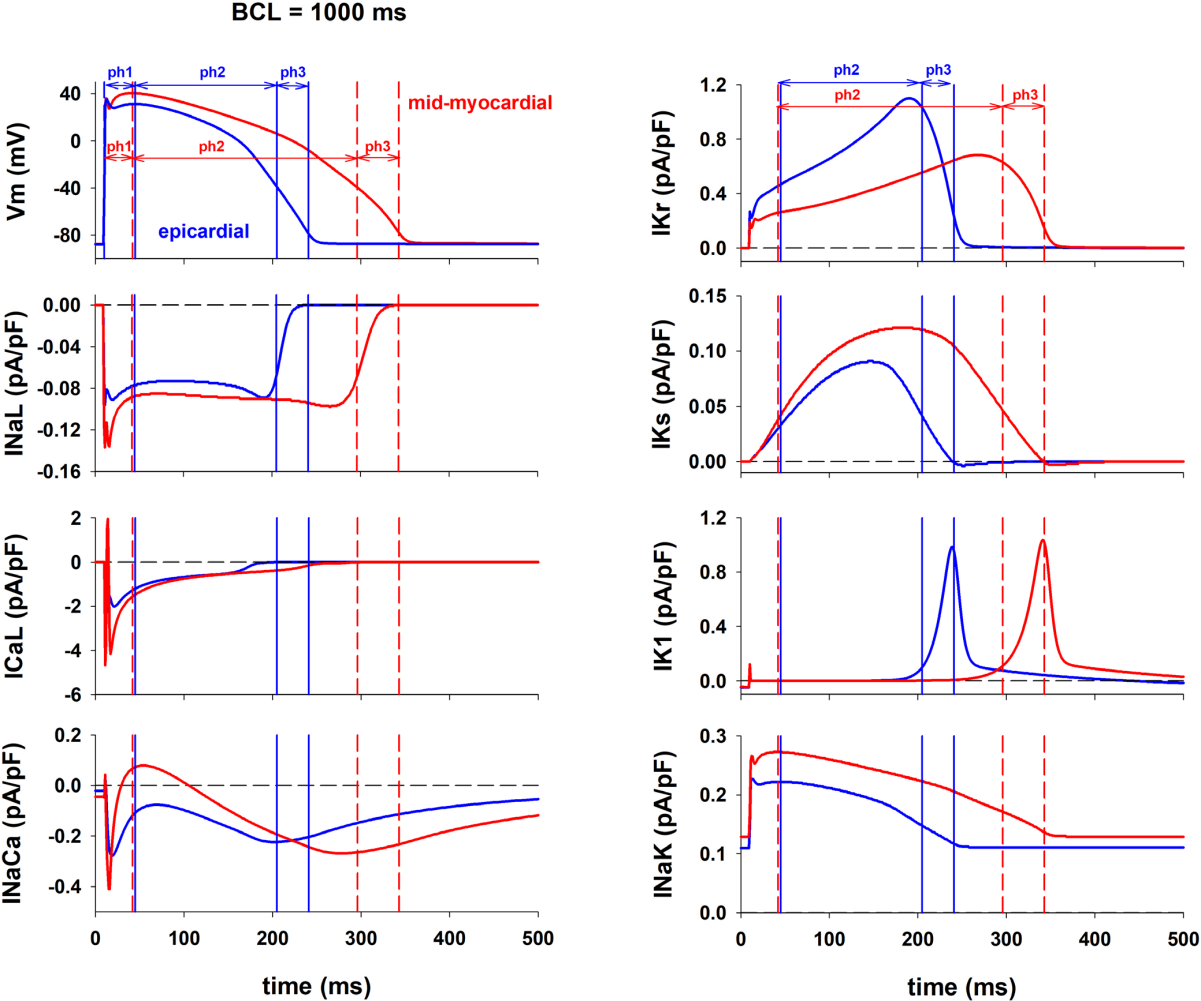
Action potentials (top left) as well as depolarizing and repolarizing ion currents during the action potential for epicardial (blue) and mid‐myocardial (red) cells during stimulation with BCL = 1000 ms during control. The different phases of the action potential are indicated for epicardial cells (vertical solid blue lines) and for mid‐myocardial cells (vertical dashed red lines). See text for detailed description.

Figure 2 shows the major depolarizing and repolarizing ion currents during the action potential for epicardial (blue) and mid‐myocardial (red) cells during stimulation with BCL = 1000 ms during control in the ORd model. Channel density of depolarizing currents, I_CaL_ and I_NaL_, in mid‐myocardial cells is about twice that of epicardial cells (O'Hara et al., 2011), which explains the larger I_CaL_ and I_NaL_ currents in mid‐myocardial cells and the more positive depolarization in the action potential of mid‐myocardial cells during phases 1 and 2 of the action potential (Figure 2, top left). The Na/Ca exchanger (I_NaCa_) depolarizing current during phase 2 and phase 3 repolarization is larger for mid‐myocardial than for epicardial cells, consistent with the 30% larger channel density of the exchanger in mid‐myocardial cells (O'Hara et al., 2011). Therefore, I_CaL_, I_NaL_, and I_NaCa_ contribute to the more positive depolarization and longer APD in mid‐myocardial than in epicardial cells. The channel density of I_Kr_ in epicardial cells is about 50% larger than in mid‐myocardial cells (O'Hara et al., 2011), which results in the much larger repolarizing current in epicardial cells (Figure 2, top right). Like I_Kr_, the channel density of I_Ks_ in epicardial cells is also about 50% larger than in mid‐myocardial cells (O'Hara et al., 2011). However, in contrast to what happened with I_Kr_, the repolarizing I_Ks_ current is larger for mid‐myocardial cells than for epicardial cells despite its smaller channel density (Figure 2, right, second plot from the top). I_Ks_ activates more slowly and at more positive transmembrane potentials than I_Kr_ (Liu & Antzelevitch, 1995). Since mid‐myocardial cells depolarize to more positive potentials and stay depolarized longer at positive potentials than epicardial cells, I_Ks_ is larger in mid‐myocardial cells (Figure 2). There are essentially no differences in I_K1_ between epicardial and mid‐myocardial cells (Figure 2, right, third plot from the top). Channel density of I_NaK_ is smaller in mid‐myocardial than in epicardial cells (O'Hara et al., 2011) but I_NaK_ current during phase 2 and phase 3 repolarization is larger in mid‐myocardial cells than in epicardial cells (Figure 2, right bottom), as a result of an increase in [Na]_i_ (from 7.8 to 8.9 mM) (Glitsch, 2001). Still, the contribution of I_Ks_ and I_NaK_ to repolarization is much smaller than that of I_Kr_ (Figure 2), and overall, the total repolarizing current is much larger in epicardial than in mid‐myocardial cells, which results in the shorter APD. In summary, the shorter APD in epicardial cells is mainly due to their larger I_Kr_, which results in a stronger total repolarizing current.

### 3.2 Rate dependence of transmural heterogeneity

**FIGURE 3:**
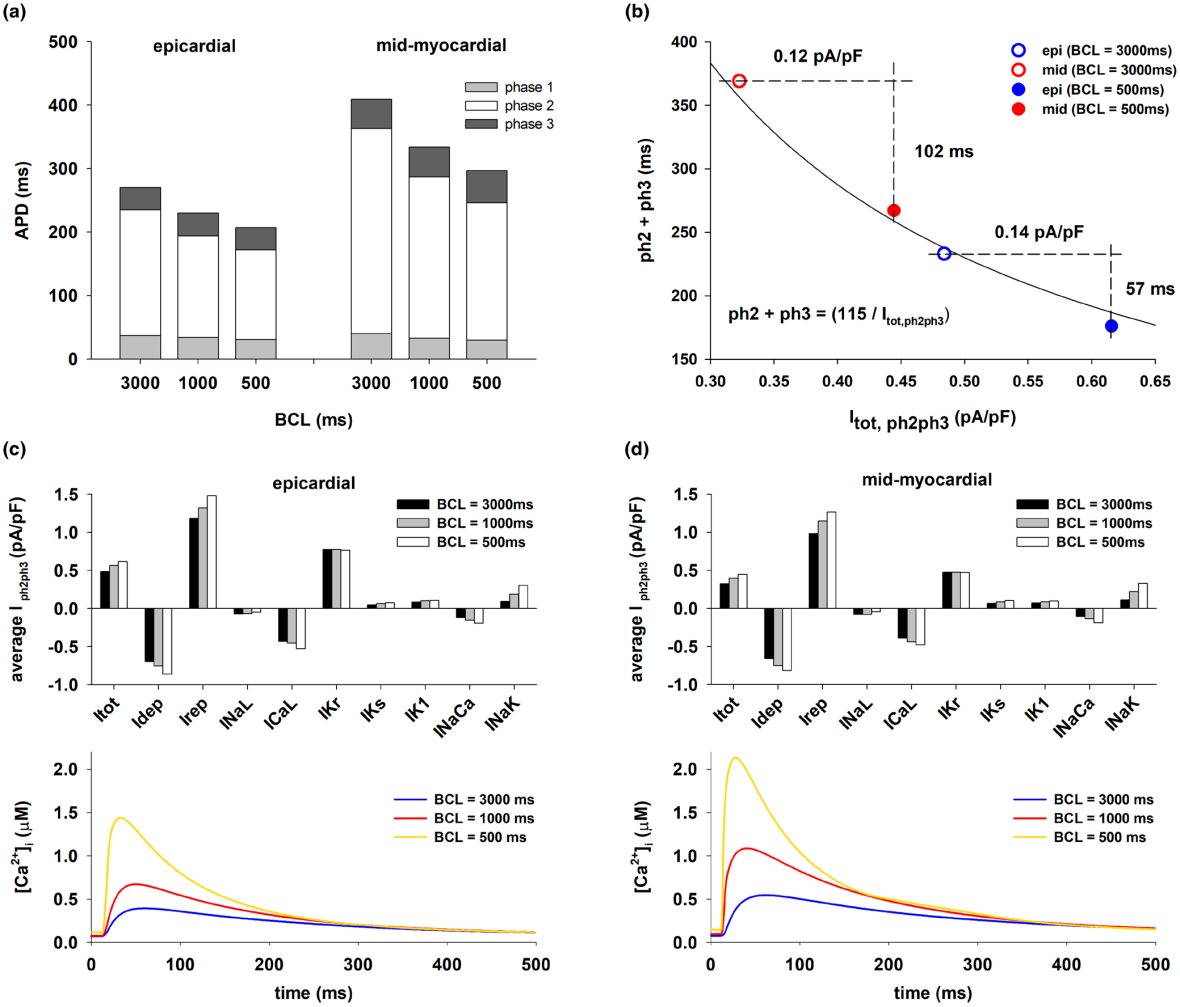
Rate dependence of dispersion of repolarization between epicardial and mid‐myocardial cells. (a) Duration of phase 1, 2, and 3 of the action potential for epicardial and mid‐myocardial cells for different BCLs. (b) Relationship between the duration of phase 2 and 3 and the average total ion current during phase 2 and 3. (c) Average ion currents during phase 2 and 3 of the action potential for epicardial cells for different BCLs. (d) Average ion currents during phase 2 and 3 of the action potential for mid‐myocardial cells for different BCLs (top); Calcium transients during the action potential for different BCLs (bottom). See text for detailed description.

**FIGURE 4:**
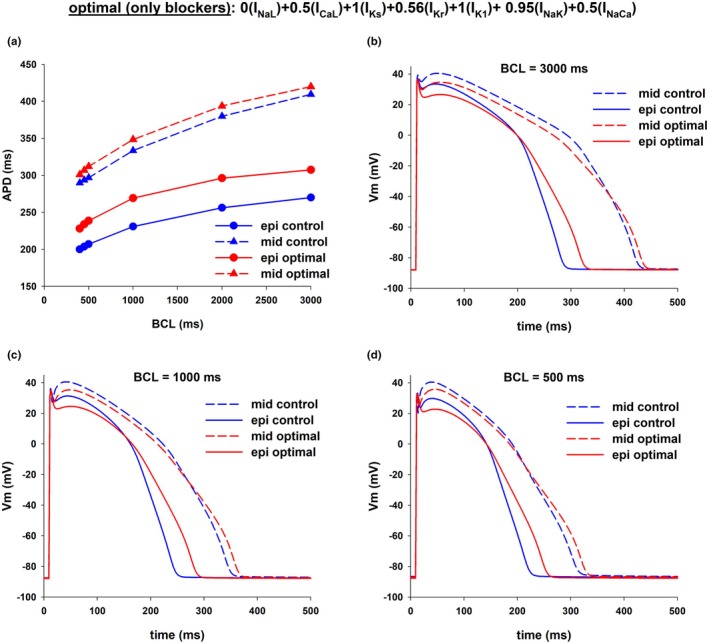
Optimal combination of ion channel blockers that results in a reduction of dispersion of repolarization while keeping APD of mid‐myocardial cells close to control. (a) The intervention reduced APD dispersion by increasing APD in epicardial cells while keeping APD in mid‐myocardial cells close to the control value by blocking several depolarizing and repolarizing currents (I_NaL_, I_CaL_, I_Kr_, I_NaK_, and I_NaCa_). Panels b–d show the corresponding action potentials of different BCLs. The format of the figure is the same as Figure 1. See text for detailed description.

Figure 1 shows that transmural heterogeneities in APD between mid‐myocardial and epicardial cells are rate dependent: a decrease in BCL leads to a decrease in APD dispersion (Figure 1a, blue solid and dashed lines). Figure 3a shows that for both epicardial and mid‐myocardial cells in the ORd model, the decrease in APD with BCL is caused by a decrease in the duration of phase 2 and 3 (i.e., the total duration of phase 2 and 3) repolarization. Figure 3b shows the relationship between average total ion current during phase 2 and 3 repolarization of the action potential (I_tot_) and the duration of phase 2 and 3 repolarization for epicardial and mid‐myocardial cells. In both cells, such a relationship can be modeled by a hyperbola, I_ion,ph2ph3_ = K/(duration of phase 2 and 3 repolarization), where K is the approximate change in transmembrane potential during phase 2 and 3 repolarization (Cabo, 2023). For both, epicardial and mid‐myocardial cells, a decrease in BCL from 3000 to 500 ms results in an increase in average I_tot_ (27% epi; 38% mid), I_dep_ (23% epi; 24% mid), and I_rep_ (25% epi; 29% mid) (black and white bars in Figure 3c,d, top). The increase in depolarizing current is a consequence of an increase in I_CaL_ and I_NaCa_ for both types of cells (I_CaL_ and I_NaCa_ in Figure 3c,d). The amplitude of the calcium transient increases as BCL is decreased (Figure 3c,d, bottom). For all BCLs the amplitude of the calcium transient is larger for mid‐myocardial than for epicardial cells (Figure 3c,d, bottom). The increase in repolarizing current is a consequence of the increase in I_NaK_ (I_NaK_ in Figure 3c,d, top). The increase in average I_tot,ph2ph3_ when BCL is reduced from 3000 to 500 ms is larger for epicardial (0.14 pA/pF) than for mid‐myocardial (0.12 pA/pF) cells (Figures 3b,c and 4d), but that increase results in a larger reduction in the duration of phase 2 and 3 for mid‐myocardial (102 ms) than for epicardial cells (57 ms). This is a consequence of the hyperbolic relationship between average total ion current during repolarization (I_tot,ph2ph3_) and the duration of phase 2 and 3 (solid line, Figure 3b); the hyperbola has a larger slope for longer values of phase 2 and 3 (mid‐myocardial cells), than for shorter values of phase 2 and 3 (epicardial cells).

### 3.3 Preferential increase of APD in epicardial cells with ion channel blockers

We used an optimization algorithm to investigate the optimal combinations of ion channel blockers that result in a reduction of APD dispersion. Figure 4 shows the results of an intervention that could reduce APD dispersion by increasing APD in epicardial cells while keeping APD in mid‐myocardial cells close to the control value by blocking several depolarizing and repolarizing currents [0(I_NaL_), 0.5(I_CaL_), 0.56(I_Kr_), 0.95(I_NaK_), and 0.5(I_NaCa_)] in the ORd model. The intervention caused a reduction of APD dispersion from 139 to 112 ms (~19% reduction) at BCL = 3000 ms, and from 90 to 73 ms (~19% reduction) at BCL = 400 ms (Figure 4a). The decrease in APD dispersion with optimal multichannel block resulted from a preferential increase in APD of epicardial cells. Figure 4b–d show the corresponding action potentials at selected BCLs. In contrast, the selective 25% block of I_Kr_, which achieves a similar prolongation of APD in epicardial cells (~40 ms) to the intervention in Figure 4, caused an 8%–9% *increase* in APD dispersion (Figure 1a). The increase in APD dispersion with the selective I_Kr_ block resulted from a preferential prolongation of APD of mid‐myocardial cells. The optimal multichannel block shown in Figure 4 decreases APD dispersion by a preferential prolongation of APD of epicardial cells.

**FIGURE 5:**
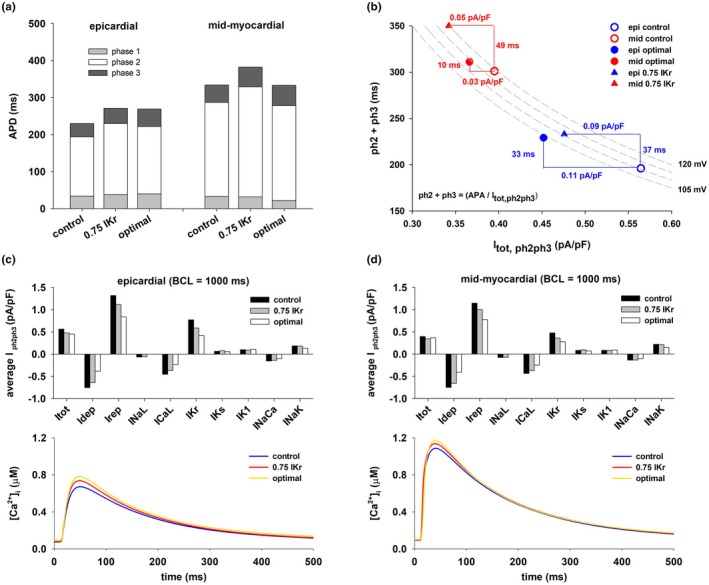
Comparison of action potential features for epicardial and mid‐myocardial cells during control, 25% block of IKr, and the optimal multichannel block (optimal) in Figure 4 that causes a reduction in dispersion of repolarization with BCL = 1000 ms. (a) Duration of phase 1, 2, and 3 for epicardial and mid‐myocardial cells. (b) Relationship between the duration of phase 2 and 3 and the average total ion current during phase 2 and 3 during control, 25% block of I_Kr_, and the optimal intervention. (c) Average ion currents during phase 2 and 3 of the action potential for epicardial cells during control, I_Kr_ block and the optimal intervention. (d) Average ion currents during phase 2 and 3 of the action potential for mid‐myocardial cells during control, I_Kr_ block and the optimal intervention (top); Calcium transients during the action potential during control, I_Kr_ block and the optimal intervention (bottom). See text for detailed description.

Figure 5 shows action potential features and average currents during phase 2 and 3 repolarization for control, selective 25% block of I_Kr_, and the optimal multichannel block [0(I_NaL_), 0.5(I_CaL_), 0.56(I_Kr_), 0.95(I_NaK_), and 0.5(I_NaCa_)] in the ORd model when BCL = 1000 ms, as shown in Figure 4. The effect of both interventions on the duration of phase 1 is much smaller than their effect on the duration of phase 2 and 3 for both types of cells (Figure 5a). Selective 25% block of I_Kr_ causes a reduction of average I_tot_ of 0.09 pA/pF in epicardial cells and of 0.05 pA/pF in mid‐myocardial cells (Figure 5b–d). The block of depolarizing currents (I_NaL_, I_CaL_, and I_NaCa_) causes about the same reduction of average depolarizing current in epicardial cells (0.11 pA/pF) and mid‐myocardial cells (0.09 pA/pF) (I_dep_ in Figure 5c,d, top). The larger reduction of average I_tot_ in epicardial cells is a consequence of the larger reduction of I_Kr_ in epicardial cells (0.18 pA/pF, Figure 5b,c) than in mid‐myocardial cells (0.11 pA/pF, Figure 5b,d). However, despite the larger reduction of I_tot_ in epicardial cells than in mid‐myocardial cells, the increase in duration of phase 2 and 3 repolarization (and APD) is smaller in epicardial than in mid‐myocardial cells. This is a consequence of the hyperbolic relationship between average I_tot_ current during phase 2 and 3 repolarization (I_tot,ph2ph3_) and the duration of phase 2 and 3 repolarization; the hyperbola has a larger slope for action potentials with longer phase 2 and 3 (mid‐myocardial cells), than for action potentials with shorter phase 2 and 3 (epicardial cells).

Optimal block of several depolarizing and repolarizing ion channels [0(I_NaL_), 0.5(I_CaL_), 0.56(I_Kr_), 0.95(I_NaK_), and 0.5(I_NaCa_)] in the ORd model increases APD in epicardial cells by 38 ms (from 231 to 269 ms) and APD of mid‐myocardial by 14 ms (from 334 to 348 ms), which results in a decrease in transmural APD dispersion from 103 to 79 ms (~23% reduction) (Figures 4c and 5a). Optimal multichannel block causes a larger reduction in average I_tot_ in epicardial cells (0.11 pA/pF) than in mid‐myocardial cells (0.03 pA/pF), resulting in the larger increase in the duration of phase 2 and 3 repolarization (and APD) in epicardial cells (Figure 5b–d). The block of depolarizing currents (I_NaL_, I_CaL_, and I_NaCa_) causes about the same reduction of average depolarizing current in epicardial cells (0.37 pA/pF) and in mid‐myocardial cells (0.34 pA/pF) (I_dep_ in Figure 5c,d). However, there is a larger decrease in average repolarizing currents in epicardial cells (0.48 pA/pF) than in mid‐myocardial cells (0.37 pA/pF) (I_rep_ in Figure 5c,d, top). That is a consequence of the larger reduction of I_Kr_ in epicardial cells (0.35 pA/pF, Figure 5b,c) than in mid‐myocardial cells (0.20 pA/pF, Figure 5b,d) and the larger contribution of I_Kr_ to the total repolarizing current in epicardial cells than in mid‐myocardial cells (I_rep_ in Figure 5c,d, top). Note that the calcium transient for both interventions (selective block of IKr, and optimal block of several depolarizing and repolarizing currents) is similar to that of control (Figure 5c,d, bottom).

### 3.4 Preferential decrease of APD in mid‐myocardial cells with ion channel blockers

**FIGURE 6:**
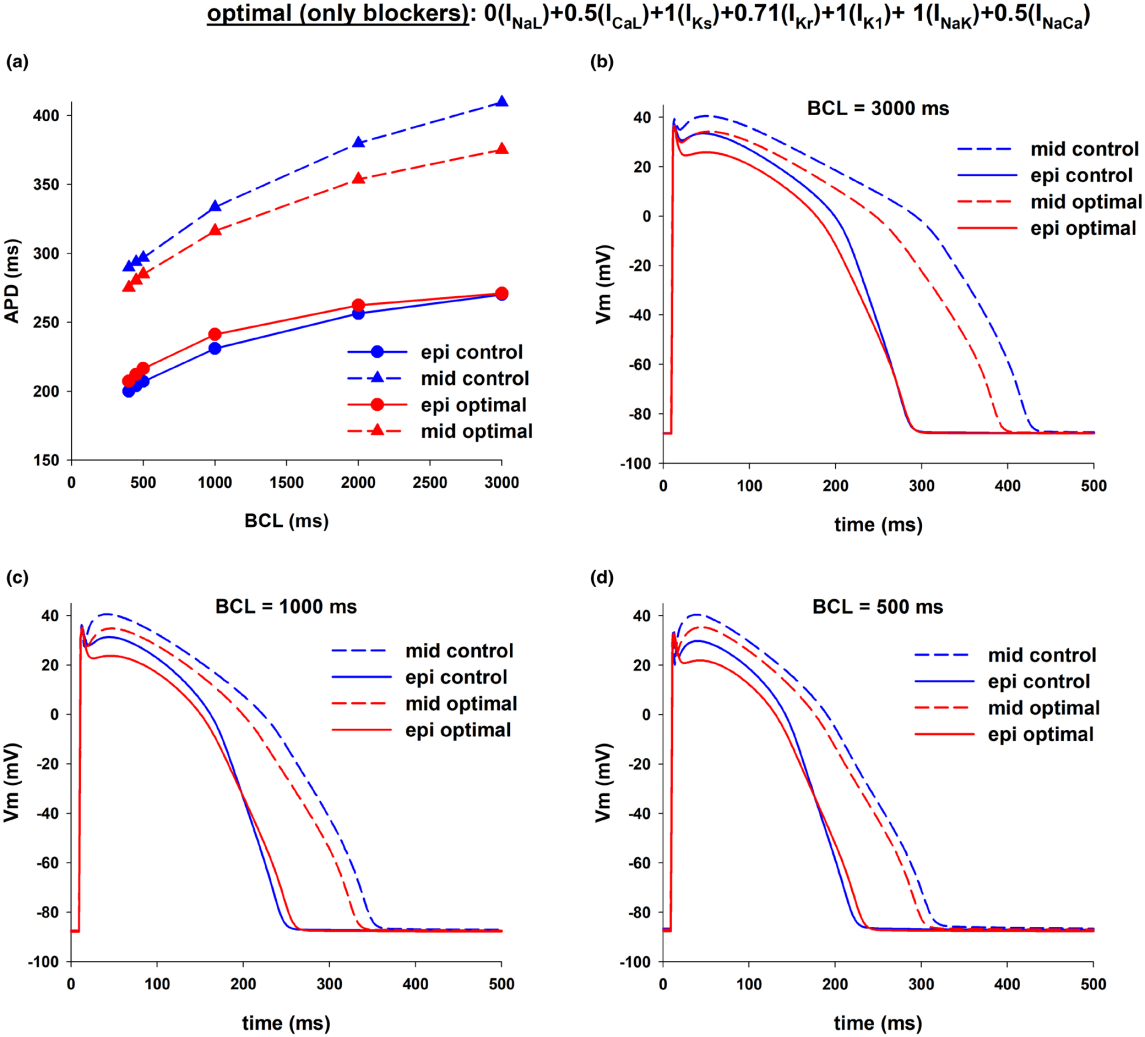
Optimal combination of ion channel blockers that results in a reduction of dispersion of repolarization while keeping APD of epicardial cells close to control. (a) The intervention reduced APD dispersion by decreasing APD in mid‐myocardial cells while keeping APD in epicardial cells close to the control value by blocking several depolarizing and repolarizing currents (I_NaL_, I_CaL_, I_Kr_, and I_NaCa_). Panels b–d show the corresponding action potentials of different BCLs. The format of the figure is the same as Figure 1. See text for detailed description.

Figure 6 shows the results of an intervention that decreases APD dispersion by reducing APD in mid‐myocardial cells while keeping APD in epicardial cells close to the control value [0(I_NaL_), 0.5(I_CaL_), 0.71(I_Kr_), and 0.5(I_NaCa_)] in the ORd model. The intervention caused a reduction of APD dispersion from 139 ms at BCL = 3000–104 ms (~25% reduction), and from 90 ms at BCL = 400–68 ms (~24% reduction) (Figure 6a). Figure 6b–d show the corresponding action potentials at selected BCLs. As with the intervention in Figure 4, the results suggest that the combined block of I_NaL_, I_CaL_, I_Kr_, and I_NaCa_ can lead to a substantial reduction in transmural APD dispersion.

### 3.5 Relative contribution of each ion current to a reduction of transmural dispersion of repolarization when using ion channel blockers

**FIGURE 7:**
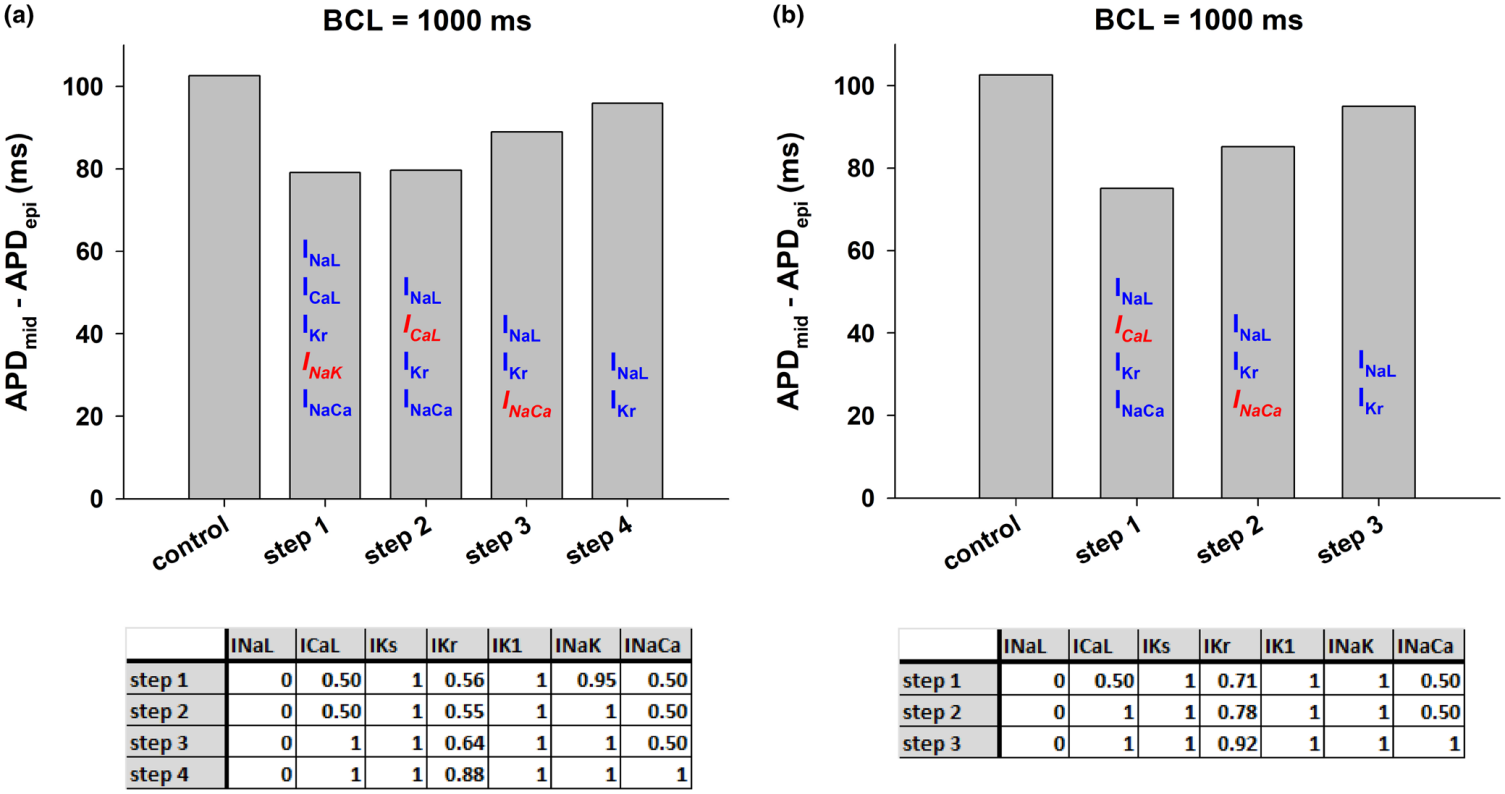
Backward feature elimination procedure applied to interventions in Figures 4 (panel a) and 6 (panel b) that reduce dispersion of repolarization when BCL = 1000 ms. The top of each panel shows APD dispersion (i.e., the difference between APD in epicardial and mid‐myocardial cells) during control and after each step in the procedure. Each vertical bar shows the ion currents that were subject to block. The ion current in red and italics indicates the current eliminated after that step in the procedure because it contributes less to reducing APD dispersion. The bottom of each panel shows the contribution of each ion current at a specific step. See text for detailed description.

To investigate the relative contribution of each ion current to the reduction of transmural APD dispersion shown in Figures 4 and 6, we used a backward feature elimination procedure (Figure 7). Figure 7a shows the results of the feature elimination procedure in the optimal multichannel block in Figure 4 on APD dispersion with BCL = 1000 ms in the ORd model. The optimal block of I_NaL_, I_CaL_, I_Kr_, and I_NaCa_ reduces APD dispersion from 103 to 79 ms (Figure 7a, top, control and step 1). Even though a 5% block of I_NaK_ is part of the optimal combination of ion channel blockers to reduce APD dispersion (Figure 7a, step 1), its elimination does not significantly change the resulting APD dispersion (Figure 7a, step 2). The elimination of I_CaL_ block (i.e., using the same I_CaL_ as control) increases APD dispersion to 89 ms (Figure 7a, step 3). Further elimination of I_NaCa_ block increases APD dispersion to 96 ms (Figure 7a, step 4). The results in Figure 7a, demonstrate the importance of I_Kr_ and I_NaL_ in the reduction of APD dispersion, but also the important contribution of I_CaL_ and I_NaCa_.

Figure 7b shows the results of the elimination procedure in the optimal multichannel block in Figure 6 on APD dispersion with BCL = 1000 ms in the ORd model. The optimal block of I_NaL_, I_CaL_, I_Kr_, and I_NaCa_ reduces APD dispersion from 103 to 75 ms (Figure 7b, top, control, and step 1). Elimination of the I_CaL_ block (i.e., using the same I_CaL_ as control) increases APD dispersion to 85 ms (Figure 7b, step 2). Further elimination of the I_NaCa_ block increases APD dispersion to 95 ms (Figure 7b, step 3). Like in Figure 7a, the results in Figure 7b indicate that I_Kr_ and I_NaL_ are the two most important ion channels to reduce APD dispersion, but it also shows that the block of I_CaL_ and I_NaCa_ contributes significantly to the reduction of APD dispersion.

### 3.6 Reduction of transmural dispersion of repolarization with ion channel blockers and activators

**FIGURE 8:**
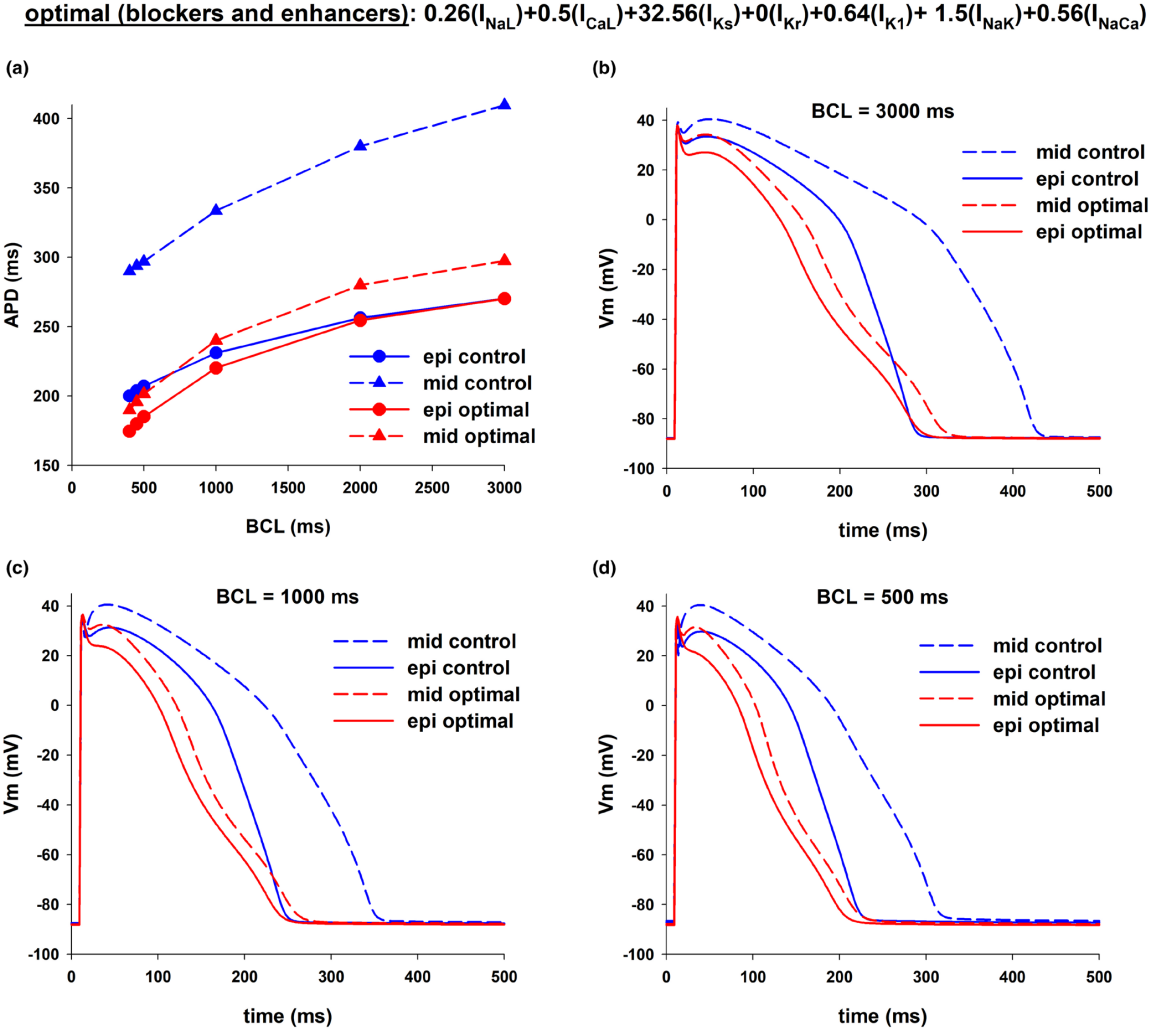
Optimal combination of ion channel blockers and activators that results in a reduction of dispersion of repolarization while keeping APD of epicardial cells close to control. (a) The intervention reduced APD dispersion by decreasing APD in mid‐myocardial cells while keeping APD in epicardial cells close to the control value by blocking and activating several depolarizing and repolarizing currents. Panels b–d show the corresponding action potentials of different BCLs. The format of the figure is the same as Figure 1. See text for detailed description.

We used an optimization algorithm to investigate the optimal combinations of ion channel blockers and activators that result in a reduction of APD dispersion. Figure 8 shows the results of an intervention that could reduce APD dispersion by reducing APD in mid‐myocardial cells while keeping APD in epicardial cells close to the control value using the ORd model. The intervention caused a reduction of APD dispersion from 139 ms at BCL = 3000–27 ms (~81% reduction), and from 90 ms at BCL = 400–16 ms (~82% reduction) (Figure 8a). Figure 8b–d show the corresponding action potentials at selected BCLs. In both epicardial and mid‐myocardial cells enhancement of I_Ks_ and block of I_Kr_ lead to an acceleration of phase 2 repolarization and a deceleration of phase 3 repolarization (Figure 8b–d). The reduction of transmural APD dispersion obtained by using an optimal combination of ion channel blockers and activators (Figure 8) is four times larger than the reduction obtained by using just ion channel blockers (Figures 4 and 6).

### 3.7 Relative contribution of each ion current to a reduction of transmural dispersion of repolarization when using ion channel blockers and activators

**FIGURE 9:**
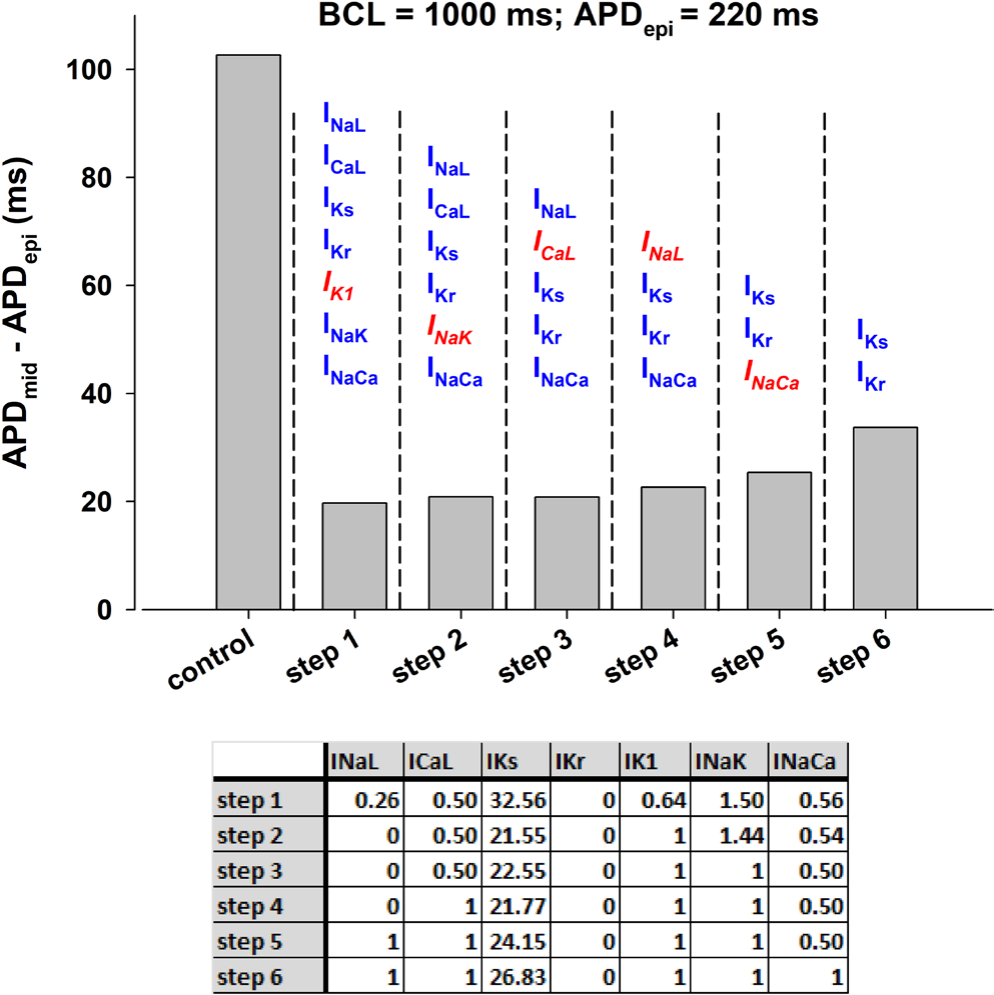
Backward feature elimination procedure applied to the intervention in Figure 8 that reduces dispersion of repolarization when BCL = 1000 ms. Top: APD dispersion (i.e., the difference between APD in epicardial and mid‐myocardial cells) during control and after each step in the procedure. The ion currents that were subject to block are shown on top of each vertical bar. The ion current in red and italics indicates the current eliminated after that step in the procedure because it contributes less to reducing APD dispersion. Bottom: Contribution of each ion current at a specific step. See text for detailed description.

To investigate the relative contribution of each ion current to the reduction of transmural heterogeneities described in Figure 8 we used a backward feature elimination procedure (Figure 9). Transmural dispersion during control, with a BCL = 1000 ms, was 103 ms (Figure 9, control). The optimal combination of ion channel conductance [0.26(I_NaL_), 0.5(I_CaL_), 32.56(I_Ks_), 0(I_Kr_), 0.64(I_K1_), 1.5(I_NaK_), (0.56)I_NaCa_] reduces APD dispersion to 20 ms (Figures 8a,c and 9, step 1). Not all ion channels contribute equally to the reduction of APD dispersion. Elimination of the modulation of I_K1_ and I_NaK_ (that is, keeping those currents the same as control) does not change the value of APD dispersion indicating that those ion channels do not contribute much to the reduction in APD dispersion (Figure 9, steps 2 and 3). Elimination of I_CaL_ increases APD dispersion modestly to 23 ms (Figure 9, step 4). Further elimination of I_NaL_ increases APD dispersion to 25 ms (Figure 9, step 5). Additional elimination of I_NaCa_ increases APD dispersion to 34 ms (Figure 9, step 6). These results show the importance of enhancing I_Ks_ and blocking I_Kr_ to obtain a strong reduction in APD dispersion; additional modulation of I_NaCa_, I_NaL_, and I_CaL_ can further decrease APD dispersion. All in all, the results in Figure 9 show that the currents that contribute the most to APD dispersion reduction are I_Ks_ and I_Kr_; modulation of just those two currents reduces APD dispersion from 103 to 34 ms at BCL = 1000 ms.

### 3.8 Mechanism of reduction of transmural dispersion of repolarization with ion channel blockers and activators

**FIGURE 10:**
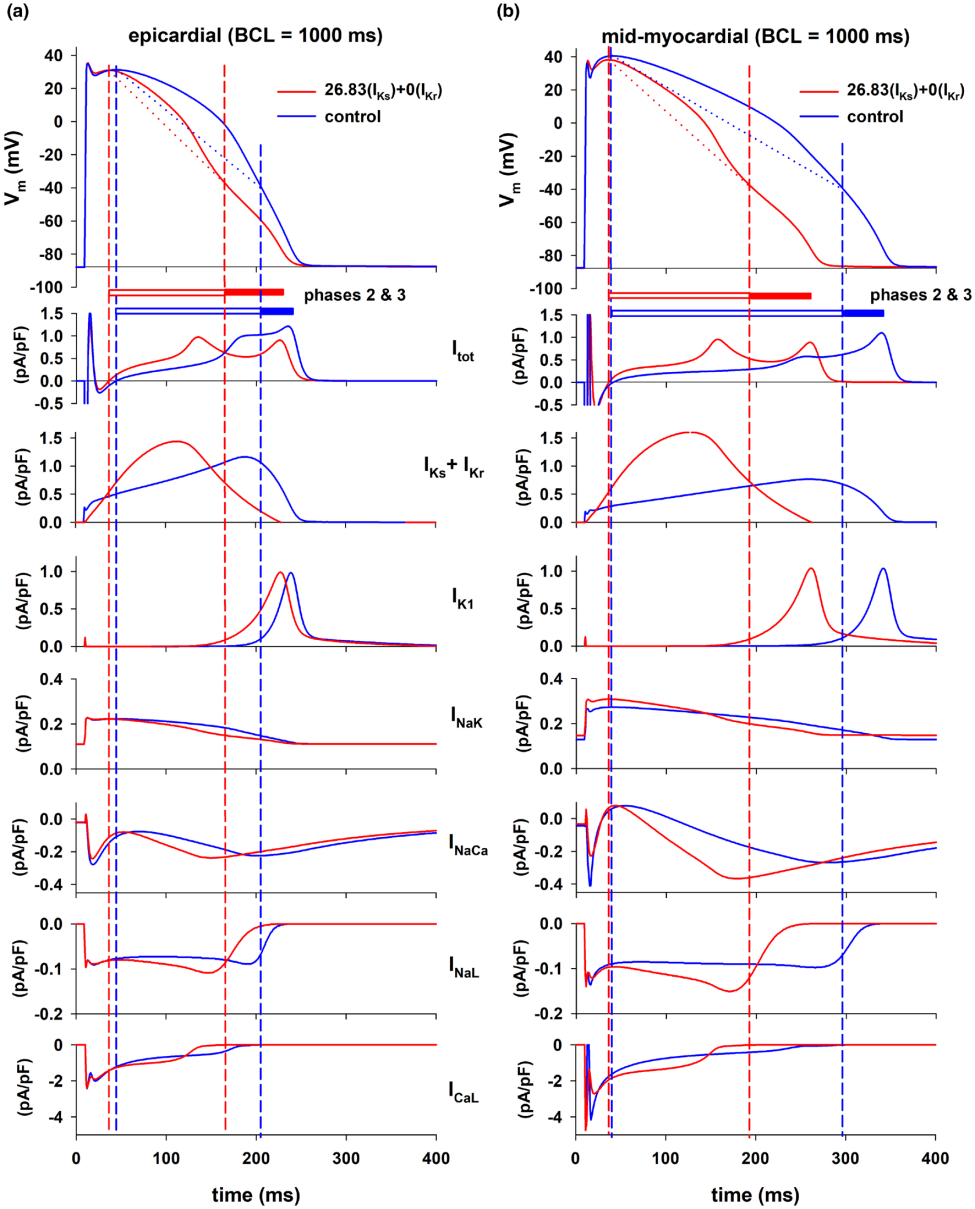
Comparison between the action potential and corresponding ion currents during control (black lines) and an intervention that activates I_Ks_ (26.83 × the control value) and blocks I_Kr_ 100% (red lines) in epicardial (panel a) and mid‐myocardial cells (panel b) when BCL = 1000 ms. Blue vertical dashed lines indicate the beginning and the end of phase 2 repolarization during control, and the red vertical dashed lines indicate the beginning and the end of phase 2 repolarization during the intervention. See text for detailed description.

Figure 9 shows that at the end of the feature elimination procedure activating I_Ks_ to 26.83× the control value and blocking I_Kr_ completely causes a strong reduction in transmural APD dispersion. That intervention results in a large shortening in mid‐myocardial cell APD and a more modest shortening in epicardial cell APD (Figure 10a,b top). To understand the mechanism of the differential APD shortening, we compared the effect on the action potential of an intervention that activates I_Ks_ and blocks I_Kr_ to the control action potential in both types of cells with BCL = 1000 ms (Figure 10). In both types of cells activating I_Ks_ (26.83× the control value) and 100% block of I_Kr_ causes a decrease in the duration of phase 2 (from 160 to 132 ms in epicardial cells and from 254 to 159 ms in mid‐myocardial cells) and an increase in the duration of phase 3 (from 36 to 61 ms in epicardial cells and from 47 to 68 ms in mid‐myocardial cells) of the action potential (Figure 10). The increase in the duration of phase 3 caused by the intervention is about the same in both cells (25 ms in epicardial and 21 ms in mid‐myocardial cells). In contrast, the decrease in the duration of phase 2 is much larger in mid‐myocardial than in epicardial cells (28 ms in epicardial vs. 95 ms in mid‐myocardial cells), which suggests that ion currents during phase 2 are responsible for the differential APD reduction caused by the modulation of the delayed rectifier currents. The difference between the average I_tot_ during control and I_K_ (= I_Kr_ + I_Ks_) modulation in phase 2 is larger in mid‐myocardial (control: 0.31 pA/pF/ms; intervention: 0.49 pA/pF/ms; slopes of blue and red dotted lines in Figure 10b, top) than in epicardial cells (control: 0.44 pA/pF/ms; intervention: 0.53 pA/pF/ms; slopes of blue and red dotted lines in Figure 10a, top). The total delayed rectifier currents are about the same in both cell types during the intervention (I_Ks_ + I_Kr_, red lines in Figure 10a,b), but they are considerably smaller in mid‐myocardial cells than in epicardial cells during control (I_Ks_ + I_Kr_, blue lines in Figure 10a,b). As a result, the difference in repolarizing currents between control and intervention is larger in mid‐myocardial than in epicardial cells. The larger difference between control and intervention in I_NaCa_, I_NaL_, and I_CaL_ (Figure 10a,b) in mid‐myocardial cells is not sufficient to compensate for the larger difference in delayed rectifier currents, which explains the larger difference in average total ionic current between control and intervention in mid‐myocardial cells, and hence the larger reduction of APD in mid‐myocardial cells. Enhancing I_Ks_ and blocking I_Kr_ results in a combined delayed rectifier current that peaks earlier in the action potential (Figure 10a,b, I_Ks_ + I_Kr_, red lines) accelerating repolarization during phase 2, and decelerating repolarization during phase 3. This results in a decrease in I_tot_ between the time (I_Ks_ + I_Kr_) peaks and the time that I_K1_ peaks (Figure 10a,b, I_tot_, red lines) leading to a non‐monotonic repolarization of the action potential in both epicardial and mid‐myocardial cells (Figure 10a,b, top, red lines).

### 3.9 Prolongation of APD without an increase in dispersion of repolarization in the ORd model

**FIGURE 11:**
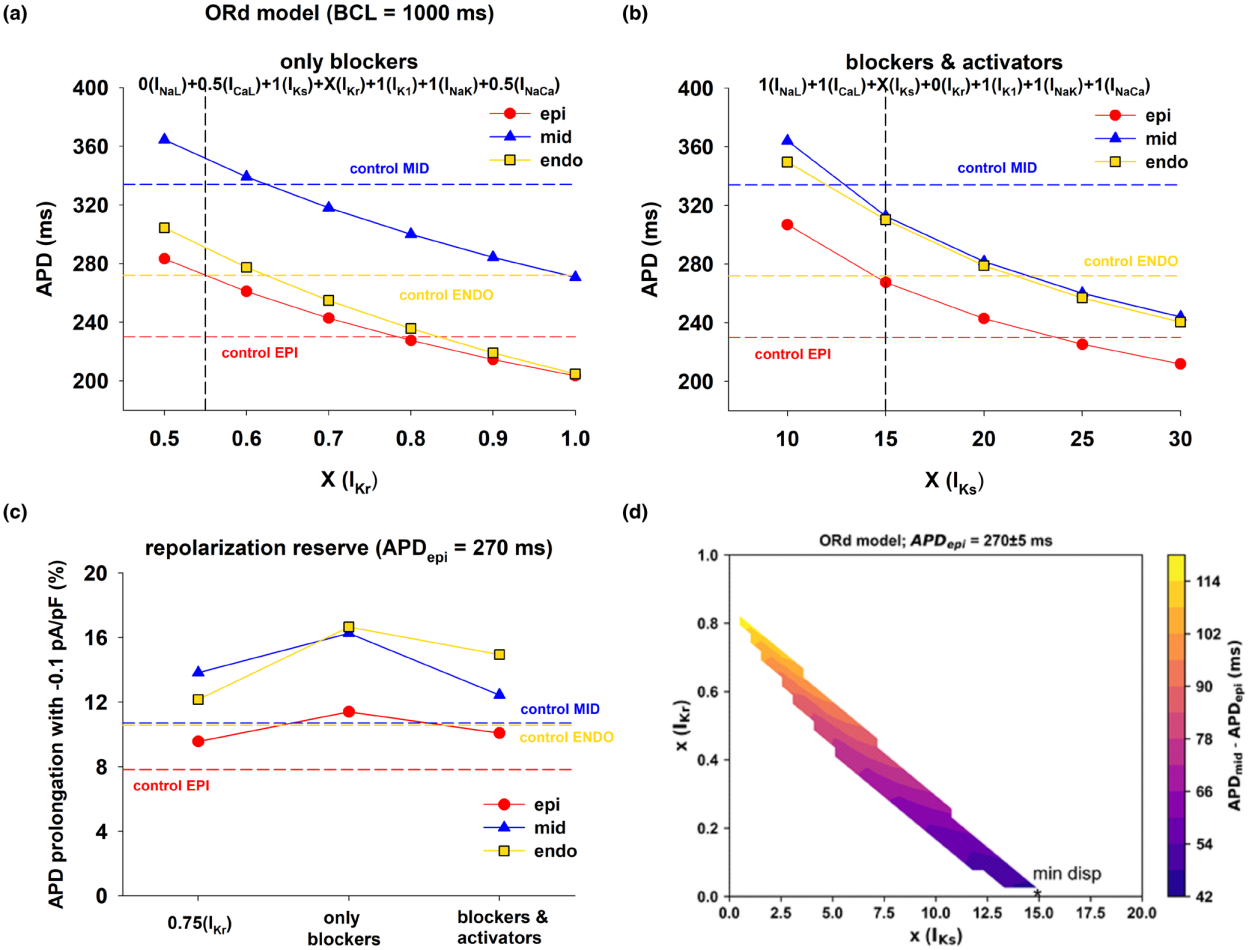
Prolongation of APD with a decrease in dispersion of repolarization in the ORd model. (a) Interventions that use only ion channel blockers. The plot shows the values of APD for epicardial (red circles), mid‐myocardial (blue triangles) and endocardial (yellow squares) cells for different levels of block of I_Kr_ (while maintaining 100% block of I_NaL_, 50% block of I_CaL_ and 50% block of I_NaCa_), with BCL = 1000 ms. (b) Interventions that use ion channel blockers and activators. The plot shows the values of APD for epicardial (red circles), mid‐myocardial (blue triangles) and endocardial (yellow squares) cells for different levels of activation of I_Ks_ (while maintaining 100% block of I_Kr_), with BCL = 1000 ms. (c) Estimation of the repolarization reserve for the different cell types and interventions. Horizontal dashed lines indicate the values of the control APD (panels a and b) and repolarization reserve during control (panel c) for epicardial (EPI), mid‐myocardial (MID) and endocardial (ENDO) cells. (d) Dispersion of repolarization (color bar) for different levels of I_Kr_ block and I_Ks_ enhancement. See text for detailed description.

In both Figures 4 and 6 the optimal combination of ion channel blockers that minimizes transmural APD dispersion in the ORd model includes 100% block of I_NaL_, 50% block of I_CaL_ and 50% block of I_NaCa_. Figure 11a shows the values of APD of epicardial (red circles), mid‐myocardial (blue triangles), and endocardial cells (yellow squares), for different levels of block of I_Kr_ (while maintaining 100% block of I_NaL_, 50% block of I_CaL_ and 50% block of I_NaCa_), with BCL = 1000 ms. For all levels of I_Kr_, transmural APD dispersion is between 70 and 80 ms (difference between mid‐myocardial and epicardial APDs in Figure 11a), which is less than APD dispersion in control (103 ms, which is the difference between the horizontal red and blue dashed lines in Figure 11a). The results in Figure 11a show that with 20%–50% block of I_Kr_ in combination with 100% block of I_NaL_, 50% block of I_CaL_ and 50% block of I_NaCa_ it is possible to prolong the APD of epicardial cells without increasing APD transmural dispersion.

Complete block of I_Kr_ and enhancement of I_Ks_ is an alternative strategy to reduce transmural APD dispersion (Figures 9 and 10). Figure 11b shows the values of APD of epicardial (red circles), mid‐myocardial (blue triangles) and endocardial cells (yellow squares), for different levels of enhancement of I_Ks_ (while maintaining 100% block of I_Kr_), with BCL = 1000 ms. For all levels of I_Ks_, APD dispersion is between 35 and 57 ms, which is less than APD dispersion in control (difference between the red and blue dashed lines in Figure 11b). The results in Figure 11b identify a second strategy for prolonging APD without increasing APD transmural dispersion by enhancing I_Ks_ (between 10 and 25× the control value) and complete block of I_Kr_.

Figure 11c shows estimations of the repolarization reserve for epicardial, mid‐myocardial and endocardial cells during control (red, blue and yellow horizontal dashed lines), and for three interventions that prolong the control epicardial APD by ~40 to ~270 ms: (1) selective 25% I_Kr_ block (Figure 1); (2) combined I_NaL_, I_CaL_, I_NaCa_, and I_Kr_ block (vertical dashed line in Figure 11a); (3) combined I_Kr_ block and I_Ks_ enhancement (vertical dashed line in Figure 11b). In Figure 11c, a *higher* number in the *y*‐axis indicates a *lower* repolarization reserve and a higher risk for early afterdepolarizations. All three interventions reduced the repolarization reserve compared to control across all cell types. In every case, epicardial cells (red circles in Figure 11c) exhibited a higher repolarization reserve than mid‐myocardial (blue triangles in Figure 11c) or endocardial (yellow squares in Figure 11c) cells. The findings also show that the repolarization reserve was greater for all cell types when both blockers and activators were used than when using blockers alone. The combined use of blockers and activators may represent a safer approach to prolong APD without increasing dispersion than the use of blockers alone.

Figure 11d shows the values of transmural dispersion (color bar) for different levels of I_Ks_ (range: 0–20× control) and I_Kr_ (range: 0–1× control) that result in action potentials with a duration of 270 ± 5 ms. A population of action potentials with epicardial cell APD = 270 ± 5 ms was generated by randomly changing the levels of I_Ks_ and I_Kr_, with all other ion currents kept at control values, using a population of models approach (Britton et al., 2013; Sobie, 2009). Figure 11d shows that modulation of I_Kr_ and I_Ks_ can be adjusted to achieve an epicardial cell APD around 270 ms with varying degrees of APD dispersion. For example, a value of 0.43 for (xI_Kr_) (i.e., 57% I_Kr_ block) and 5 for (xI_Ks_) (i.e., enhancing the value of I_Ks_ to 5× control) results in an APD = 271 ms, with a transmural dispersion of 77 ms, which is not the minimal possible (Figure 11d, asterisk labeled as “min disp”), but it is less than control (103 ms).

### 3.10 Prolongation of APD without an increase in dispersion of repolarization in the ToR‐ORd model

**FIGURE 12:**
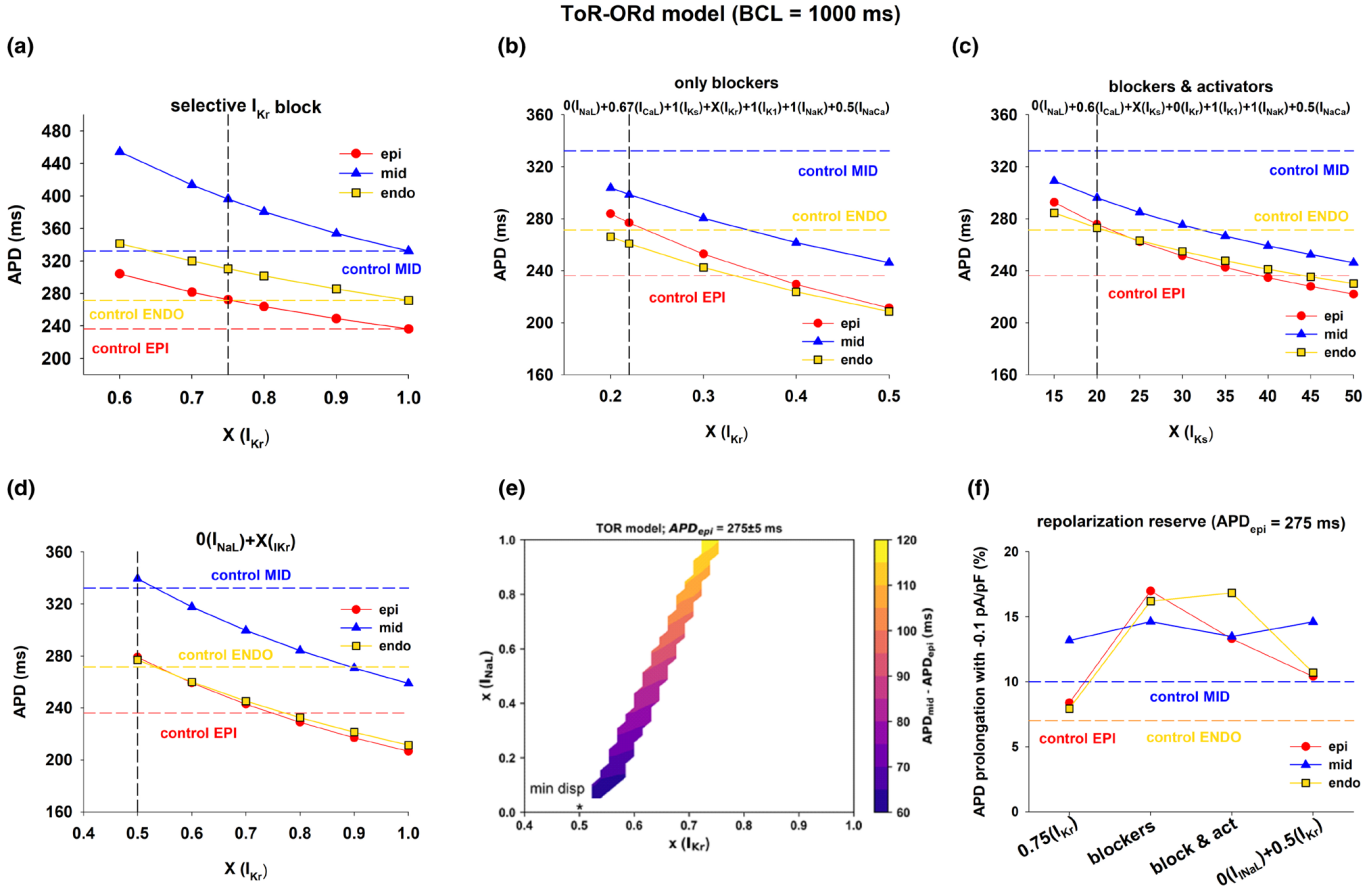
Prolongation of APD with a decrease in dispersion of repolarization in the ToR‐ORd model. (a) Interventions using selective I_Kr_ blockers. The plot shows the values of APD for epicardial (red circles), mid‐myocardial (blue triangles), and endocardial (yellow squares) cells for different levels of selective block of I_Kr_, with BCL = 1000 ms. (b) Interventions that use only ion channel blockers. The plot shows the values of APD for epicardial (red circles), mid‐myocardial (blue triangles), and endocardial (yellow squares) cells for different levels of block of I_Kr_ (while maintaining 100% block of I_NaL_, 33% block of I_CaL_, and 50% block of I_NaCa_), with BCL = 1000 ms. (c) Interventions that use ion channel blockers and activators. The plot shows the values of APD for epicardial (red circles), mid‐myocardial (blue triangles) and endocardial (yellow squares) cells for different levels of activation of I_Ks_ (while maintaining 100% block of I_NaL_, 40% block of I_CaL_, 100% block of I_Kr_, and 50% block of I_NaCa_), with BCL = 1000 ms. (d) Values of APD for epicardial (red circles), mid‐myocardial (blue triangles) and endocardial (yellow squares) cells with 100% block of I_NaL_ for different levels of block of I_Kr_, with BCL = 1000 ms. (e) Dispersion of repolarization (color bar) for different levels of I_NaL_ and I_Kr_ block. (f) Estimation of the repolarization reserve for the different cell types and interventions. Horizontal dashed lines indicate the values of the control APD (panels a–d) and repolarization reserve during control (panel f) for epicardial (EPI), mid‐myocardial (MID), and endocardial (ENDO) cells. See text for detailed description.

We tested if the results obtained with the ORd model (Figures 1, 2, 3, 4, 5, 6, 7, 8, 9, 10, 11) could be reproduced with the ToR‐ORd model of the action potential of human ventricular cells, using similar protocols. Figure 12a shows APDs of epicardial (red circles), endocardial (blue triangles), and mid‐myocardial (yellow squares) cells for different levels of selective I_Kr_ block. The results indicate that selective block of I_Kr_ results in APD prolongation with an increase in APD transmural dispersion because prolongation in mid‐myocardial cells is larger than in epicardial cells, similar to what occurred in the ORd model (Figure 1). For example, a 25% block of I_Kr_ results in epicardial cell APD prolongation from 236 to 272 ms (15% increase), and mid‐myocardial cell prolongation from 332 to 396 ms (19% increase), with BCL = 1000 ms (Figure 12a, vertical dashed line). With that intervention, APD dispersion increased from 96 ms (difference between red and blue horizontal dashed lines labeled as “control EPI” and “control MID” in Figure 12a) to 124 ms (APD difference between red circle and blue triangle on the vertical dashed line).

Figure 12b shows the values of APD of epicardial (red circles), mid‐myocardial (blue triangles) and endocardial cells (yellow squares), for different levels of block of I_Kr_, while maintaining 100% block of I_NaL_, 33% block of I_CaL_, and 50% block of I_NaCa_, with BCL = 1000 ms. The optimal combination of ion channel blockers to minimize APD dispersion between epicardial and mid‐myocardial cells was obtained using the same optimization algorithm used in the ORd model. The results are consistent with those obtained in the ORd model (Figure 11a) indicating that block of I_Kr_ in combination with block of I_NaL_, I_CaL_, and I_NaCa_ prolongs epicardial cell APD without increasing APD transmural dispersion. For example, a 78% block of I_Kr_ (i.e., 0.22× I_Kr_) results in an APD prolongation from 236 to 277 ms at BCL = 1000 ms (Figure 12b, vertical dashed line). With that intervention, APD dispersion decreased from 96 ms (difference between red and blue horizontal dashed lines labeled as “control EPI” and “control MID” in Figure 12b) to 38 ms (APD difference between yellow square and blue triangle on the vertical dashed line). The reduction of APD dispersion in the ToR‐ORd model using channel blockers was 60% (from 96 to 38 ms), larger than in the ORd model which was 22% (from 103 to 80 ms). A difference between the ORd and ToR‐ORd models is that for the intervention in Figure 12b, transmural dispersion is determined by the difference between mid‐myocardial and endocardial cells, instead of the difference between mid‐myocardial and epicardial cells (Figure 11a).

Figure 12c shows the values of APD of epicardial (red circles), mid‐myocardial (blue triangles), and endocardial cells (yellow squares), for different levels of enhancement of I_Ks_, while maintaining 100% block of I_NaL_, 40% block of I_CaL_, 100% block of I_Kr_, and 50% block of I_NaCa_, with BCL = 1000 ms. As occurred with the ORd model, I_Kr_ block, and I_Ks_ enhancement further reduce APD transmural dispersion. For example, a 20× enhancement of IKs (i.e., 20× I_Ks_) results in an APD prolongation from 236 to 275 ms at BCL = 1000 ms (Figure 12c, vertical dashed line). With that intervention, APD dispersion decreased from 96 ms (difference between red and blue horizontal dashed lines labeled as “control EPI” and “control MID” in Figure 12c) to 23 ms (APD difference between yellow square and blue triangle on the vertical dashed line) (~76% reduction). The reduction in APD dispersion in the ToR‐ORd model using blockers and enhancers was achieved by blocking not only I_Kr_, but also blocking I_NaL_, I_CaL_, and I_NaCa_ (Figure 12c). In contrast, with the ORd model, a significant reduction in APD dispersion could be achieved with 100% block of I_Kr_ and enhancement of I_Ks_, without modulation of any other currents (Figure 11b).

When using ion channel blockers and enhancers, the two most important currents to decrease APD dispersion in the ORd model were I_Kr_ and I_Ks_ (Figure 9). In the ToR‐ORd model, the backward feature elimination algorithm identified I_NaL_ and I_Kr_ as the two most important currents to decrease APD dispersion. APDs for different cell types with 100% block of I_NaL_ and different levels of I_Kr_ block are shown in Figure 12d. For a prolongation of the APD of epicardial cells to 278 ms (dashed vertical line in Figure 12d), APD dispersion is reduced from the control value of 96– 62 ms (~35% reduction). Also note that for X(I_Kr_) = 1 (that is when cells are only subjected to 100% block of I_NaL_), the shortening of APD in mid‐myocardial cells (22%) is larger than the shortening of APD in epicardial cells (12%), indicating that the block of depolarizing currents has a larger effect in shortening APD in mid‐myocardial cells than in epicardial cells.

Figure 12e shows the values of transmural dispersion (color bar) for different levels of I_NaL_ and I_Kr_ block that result in epicardial action potentials with a duration of 275 ± 5 ms. The minimal APD dispersion is indicated by the asterisk (labeled as “min disp”). A population of action potentials with epicardial cell APD = 275 ± 5 ms was generated by randomly changing the levels of I_NaL_ and I_Kr_, with all other ion currents kept at control values. Figure 12e shows that modulation of I_Kr_ and I_Ks_ can be adjusted to achieve an epicardial cell APD around 275 ms with varying degrees of APD dispersion.

Figure 12f shows estimations of the repolarization reserve for epicardial, mid‐myocardial and endocardial cells during control (red, blue and yellow horizontal dashed lines), and for four interventions that prolong the control epicardial APD to ~275 ms: (1) selective 25% I_Kr_ block (vertical dashed line in Figure 12a); (2) combination of ion channel blockers (vertical dashed line in Figure 12b); (3) combination of ion channel blockers and enhancers (vertical dashed line in Figure 12c); (4) 100% block of I_NaL_ and 50% block of I_Kr_ (vertical dashed line in Figure 12d). All interventions resulted in a decrease of the repolarization reserve with respect to control. For interventions that result in a larger reduction in APD dispersion (Figure 12f, blockers, blockers and activators) the repolarization reserve is more diminished than for interventions with a more modest reduction in APD dispersion (Figure 12f, 0(I_NaL_) + 0.5(I_Kr_)). Overall, the decrease in the repolarization reserve for interventions that reduce APD dispersion are similar in the ORd (Figure 11c) and ToR‐ORd models (Figure 12f).

## 4 DISCUSSION

Class III antiarrhythmic drugs primarily act by blocking potassium channels, leading to APD prolongation to prevent reentrant arrhythmias (Peters et al., 2000). However, as a result of the heterogeneity in cell types across the myocardial wall, agents that increase APD by selectively blocking repolarizing potassium currents like I_Kr_, may increase APD dispersion, which has been shown to be pro‐arrhythmic (Antzelevitch, 2005, 2008; Avula et al., 2019; Kuo et al., 1985; Surawicz, 1989). In this report, using computer models of the action potential of human epicardial, mid‐myocardial and endocardial myocytes, we have identified two strategies to prolong APD without increasing transmural APD dispersion. The first strategy involves blocking several depolarizing and repolarizing ionic currents. Block of I_NaL_, I_CaL_, I_Kr_, and I_NaCa_ can increase APD in epicardial cells while reducing APD dispersion during control by about 20%–60%, depending on the model (Figures 11a and 12b). The second strategy involves the use of a combination of ion channel blockers and activators which results in prolongation of APD in epicardial cells with a stronger reduction in transmural APD dispersion than using only ion channel blockers (Figures 11b and 12c).

Most of the evidence in the literature suggests that selective I_Kr_ block increases transmural APD dispersion because I_Kr_ blockers preferentially prolong the action potential of mid‐myocardial cells (Antzelevitch, 2008; Lukas, 1997). This increase in dispersion may provide a substrate for the initiation and maintenance of reentrant arrhythmias like TdP (Antzelevitch, 2005). Examples include class III selective I_Kr_ blockers like dofetilide and sotalol (Antzelevitch, 2008; Lukas, 1997). The results of our computer simulations, with both the ORd and ToR‐ORd models, are consistent with those experimental and clinical findings: a 25% block of I_Kr_ prolongs preferentially the APD of mid‐myocardial cells, increasing APD dispersion by 8%–9% in the ORd model (Figure 1) and by 29% in the ToR‐ORd model (Figure 12a). Despite the larger reduction of average I_tot_ in epicardial cells than in mid‐myocardial cells with a 25% block of I_Kr_, the increase in duration of phase 2 and 3 repolarization (and consequently APD) is smaller in epicardial than in mid‐myocardial cells (Figure 5b). This is a consequence of the hyperbolic relationship between average I_tot_ current during phase 2 and 3 repolarization (I_tot,ph2ph3_) and the duration of phase 2 and 3 repolarization; the hyperbola has a larger slope for action potentials with longer phase 2 and 3 (mid‐myocardial cells), than for action potentials with shorter phase 2 and 3 (epicardial cells).

While selective I_Kr_ blockers generally increase transmural APD dispersion, drug agents that block multiple channels in addition to I_Kr_ have been shown to decrease (or at least not to increase) transmural dispersion. For example, ranolazine, which blocks I_NaL_ and I_CaL_ in addition to I_Kr_, has been shown to decrease transmural dispersion (Antzelevitch et al., 2004). Also, the reduction in I_NaL_ leads to a decrease in intracellular sodium levels which in turn reduces the reverse mode of I_NaCa_ (Belardinelli et al., 2006). Similarly, amiodarone, which is a multichannel acting agent that blocks potassium (I_Kr_, I_Ks_, and possibly I_K1_), sodium (I_Na_ and I_NaL_), calcium (I_CaL_) channels (Árpádffy‐Lovas et al., 2021; Drouin et al., 1998; Gelman et al., 2024; Sicouri et al., 1997; Vassallo & Trohman, 2007) as well as the sodium/calcium exchanger (Watanabe & Kimura, 2000), has also been shown to reduce dispersion of repolarization. In that context, our findings show that optimal multichannel block of I_NaL_, I_CaL_, I_Kr_, and I_NaCa_ can reduce APD dispersion by about 20% in the ORd model (Figures 4, 6, 7, and 11A) and by 60% in the ToR‐ORd model (12B) and are consistent with available experimental and clinical evidence. Figure 11a shows that for an intervention that only blocks depolarizing currents (I_NaL_, I_CaL_, and I_NaCa_), while keeping I_Kr_ at the same value as control (abscissa = 1.0 in Figure 11a), the reduction of APD is more pronounced for mid‐myocardial cells (from 334 to 271 ms) than for epicardial cells (from 231 to 204 ms) thus reducing APD dispersion (from 103 to 67 ms). Therefore, while block of repolarizing current I_Kr_ prolongs preferentially APD in mid‐myocardial cells (Figure 1), block of depolarizing currents (100% block of I_NaL_, 50% block of I_CaL_ and 50% block of I_NaCa_) shortens preferentially APD in mid‐myocardial cells (Figure 11a). The same effects also occur in the ToR‐ORd model: block of repolarizing currents preferentially prolongs APD in mid‐myocardial cells (Figure 12a), and block of depolarizing currents preferentially shortens APD in mid‐myocardial cells (Figure 12d). All in all, the contrasting and balancing effect of blocking depolarizing and repolarizing ion currents in epicardial and mid‐myocardial cells makes it possible to design an optimal strategy to prolong APD without an increase in APD dispersion using multichannel blockers. This mechanism may explain how drugs like ranolazine and amiodarone prolong APD without an increase in APD dispersion by blocking several depolarizing and repolarizing ion channels.

The results in Figure 11b show that, in the ORd model, 100% block of I_Kr_ and enhancement of I_Ks_ can increase APD of epicardial cells while reducing transmural APD dispersion by ~70% (Figure 9). In the ToR‐ORd model complete block of I_Kr_ and enhancement of I_Ks_, along with block of I_NaL_, I_CaL_, and I_NaCa_, also caused a reduction of transmural APD dispersion by ~70% (Figure 12c); however, block of I_Kr_ and enhancement of I_Ks_ alone was not sufficient for a reduction in APD dispersion. Transmural APD dispersion between epicardial and mid‐myocardial cells is mainly the result of the larger I_Kr_ in epicardial cells (Figure 2), which results in a stronger total repolarizing current. The contribution of I_Ks_ to repolarization in both epicardial and mid‐myocardial cells is much smaller than that of I_Kr_ (Figure 2). Interestingly, despite its smaller channel density, during the action potential, I_Ks_ is larger for mid‐myocardial cells than for epicardial cells (Figure 2). This is a consequence of the morphology of the action potential and the dynamics of activation of I_Ks_. I_Ks_ activates more slowly and at more positive transmembrane potentials than I_Kr_ (Liu & Antzelevitch, 1995), and since mid‐myocardial cells depolarize to more positive potentials and stay depolarized longer at positive potentials than epicardial cells, I_Ks_ is larger in mid‐myocardial than in epicardial cells (Figure 2). Therefore, it is expected that shifting the responsibility of repolarization from I_Kr_ to I_Ks_ would result in a decrease of APD dispersion. Our results are consistent with a computational study by Christophe (2015) who observed that enhancement of I_Ks_ activity results in a decrease in transmural APD dispersion. However, there is experimental evidence suggesting that enhancing I_Ks_ can lead to an increased transmural APD dispersion. Mutations in KCNQ1, which encodes for KvLQT1 a component of I_Ks_ can lead to Short QT Syndrome (SQTS) due to a gain of function in I_Ks_. This gain of function can cause a heterogeneous abbreviation of APD and refractoriness, and an increased inducibility of ventricular fibrillation as a result of an increased APD dispersion (Milberg et al., 2007). Kang et al. (2017) found that in left ventricle wedge preparations from explanted human hearts, beta‐adrenergic stimulation can significantly increase I_Ks_ and its contribution to human left ventricular repolarization, as well as conduction velocity. Moreover, Kang et al. (2017) reported that beta‐adrenergic stimulation combined with I_Kr_ channel blocker E‐4031 led to a significant increase in transmural dispersion of repolarization. In our simulations I_Ks_ enhancement and I_Kr_ block decrease transmural dispersion (Figure 11b,d). Discrepancies between the experimental findings and the numerical results may be attributed to factors influencing transmural dispersion of repolarization in myocardial tissue, such as cell‐to‐cell coupling, cardiac conduction (Kang et al. (2017) report that conduction velocity increases with beta‐adrenergic stimulation) and APD gradients (Glukhov et al., 2010).

The magnitude of I_Ks_ can be increased multiple fold through various regulatory mechanisms, primarily involving sympathetic stimulation and intracellular signaling molecules as well as by increasing protein trafficking to the cell membrane. Beta‐adrenergic stimulation with 30 nM isoproterenol can increase I_Ks_ about 10‐fold and reverse the importance of I_Ks_ and I_Kr_ for repolarization in guinea pig hearts (Banyasz et al., 2014). Thompson et al. (2017) reported a 2.5‐fold increase in I_Ks_ during cAMP stimulation as a result of an increase in the likelihood of channel opening. Li et al. (2013) showed that an increase in intracellular ATP concentration can result in a 3 to 5‐fold increase in I_Ks_. Jiang et al. (2017) showed that, under conditions of stress, KCNQ1 can traffic from intracellular reservoirs to the cell membrane to increase the magnitude of I_Ks_. Additionally, I_Ks_ activators can increase the magnitude of the current by around 2‐fold (Bohannon et al., 2020; Xu et al., 2002, 2015). In summary, the evidence suggests that the maximal I_Ks_ increase that can be achieved experimentally is about 10‐fold, which is smaller than the 15‐fold (Figure 11b) and 20‐fold (Figure 12c) I_Ks_ increase necessary to minimize APD dispersion in the computer models, indicating that the minimal APD dispersion with I_Ks_ activators may not be achievable. On the other hand, it is possible that the presence of cells with different APDs in the ventricular wall can create a transmural dispersion that can be beneficial to synchronize repolarization preventing rhythm disturbances and improving contraction. Therefore, when developing a strategy to increase APD, minimizing APD dispersion may neither be achievable nor desirable. As shown in Figures 11d and 12e, for a given prolongation of APD, by adjusting the degree of enhancement or inhibition of ion currents, it is possible to obtain any value of APD dispersion between control and the minimal possible.

In conclusion, there is agreement between published experimental and clinical results and the numerical simulations presented in this report indicating that interventions that block several depolarizing and repolarizing ion channels important during phase 2 and 3 of the action potential can prolong APD without increasing or even reducing transmural APD dispersion. The numerical simulations in this report provide insight into the mechanisms of that reduction, which ultimately stems from the larger effect of changes in total average ion current (caused by block of depolarizing and repolarizing ion channels) in the APD of mid‐myocardial cells than in the APD of epicardial cells. This differential effect is a consequence of the hyperbolic relationship between average total ion current and the duration of phase 2 and 3 repolarization and can be used to reduce APD dispersion while increasing APD. Conversely, there is no direct experimental or clinical evidence indicating that the second strategy proposed in this report, I_Kr_ blockade in combination with I_Ks_ activation, reduces transmural APD dispersion. The two strategies presented here rely on block of I_Kr_, but there is strong experimental and clinical evidence showing that selective I_Kr_ block leads to an increase in APD dispersion. Therefore, I_Kr_ block should be accompanied by interventions in other ion channels, either by blocking depolarizing currents (first strategy) or activating other repolarizing currents (second strategy).

### 4.1 Limitations

Computer models of the action potential integrate, often conflicting, experimental data obtained under different conditions from different preparations. As a consequence, conclusions derived from numerical simulations of the cardiac action potential should be interpreted with caution and the predictions need to be tested in single cell experiments. In this report we simulate the effect of anti‐arrhythmic agents on ion channels by modulating the maximum channel conductance (see Section 2). However, pharmacological agents have binding and unbinding kinetics which may change at different stimulation rates, adding another layer of complexity to their modulation of the channel which was not considered in this report. Moreover, during the propagation of the action potential in myocardial tissue, electrotonic interaction between neighboring cells can modulate the effects of enhancing or inhibiting ion currents observed in single cells (Decker et al., 2009). In this report we estimated transmural dispersion of repolarization as the maximum difference between APDs between epicardial, mid‐myocardial and endocardial cells. But transmural dispersion of repolarization in myocardial tissue is also affected by cell‐to‐cell coupling, cardiac conduction and APD gradients (Glukhov et al., 2010). Therefore, the conclusions derived from this study will have to be confirmed in experimental studies in single cells as well as in myocardial tissue.

## FUNDING INFORMATION

This work was supported in part by PSC‐CUNY Award # 67024‐0055.

## CONFLICT OF INTEREST STATEMENT

The author declares that there is no conflict of interest.

## ETHICS STATEMENT

This study does not require ethical approval.
